# High-order spoof localized surface plasmons supported on a complementary metallic spiral structure

**DOI:** 10.1038/srep24447

**Published:** 2016-04-15

**Authors:** Zhen Gao, Fei Gao, Baile Zhang

**Affiliations:** 1 Division of Physics and Applied Physics, School of Physical and Mathematical Sciences, Nanyang Technological University, Singapore 637371, Singapore; 2 Centre for Disruptive Photonic Technologies, Nanyang Technological University, Singapore 637371, Singapore

## Abstract

We experimentally demonstrate that multiple high-order spoof localized surface plasmons (spoof-LSPs) modes can be supported on a complementary metallic spiral structure, which were absent in the previously reported spoof-LSPs modes. Through exact numerical simulations and near-field imaging experiments, we directly observe these high-order spoof-LSPs modes at microwave frequencies. We also show that these higher-order spoof-LSPs modes exhibit larger frequency shifts caused by the local environmental refractive index change than the previously reported low-order spoof-LSPs modes. Hence the complementary MSS may find potential applications as plasmonic sensor in the microwave and terahertz frequencies.

Surface plasmons (SPs), as coherent electronic oscillations at the interfaces between metals and dielectric materials, have been intensively studied in recent years^1^,^2^. Surface plasmons exist either as propagating surface plasmon polaritons (SPPs) for extended interfaces and waveguide configuration or as localized surface plasmons (LSPs) in finite structures, i.e., plasmonic particles^3^. Recently, to transfer the exciting properties of propagating surface plasmon polaritons (SPPs) and localized surface plasmons (LSPs) to low frequencies (far infrared, terahertz, and microwave), the concept of spoof (or designer) surface plasmons (spoof-SPPs)^4^,^5^,^6^,^7^,^8^,^9^,^10^,^11^ and spoof localized surface plasmons (spoof-LSPs) were developed^12^,^13^,^14^,^15^,^16^ consequently. Particularly, a deep-subwavelength metallic spiral structure (MSS)^17^,^18^ and its Babinet-inverted (complementary) structure^19^ can support magnetic spoof-LSPs (magnetic dipole mode). Though high-order electric/magnetic spoof-LSPs modes have been experimentally observed on MSS^18^, similar high-order electric/magnetic spoof-LSPs modes have not been observed on complementary MSS. It is interesting if there also exist high-order modes of spoof-LSPs on complementary MSS following the Babinet’s principle^20^. Moreover, while electric high-order spoof-LSPs modes in both azimuthal^13^,^14^ and radial directions^16^ have been widely observed, high-order modes of magnetic spoof-LSPs on complementary MSS still remain unclear and thus deserve a detailed examination. For the development of plasmonic devices, a detailed knowledge of the characteristic of spoof-LSPs is essential. The high-order modes of spoof-LSPs existing on the complementary MSS are possible to provide multiple resonance peaks, which obviously facilitate the application of spoof-LSPs in the microwave or terahertz domains.

In this report, we show that complementary MSS can also support multiple high-order spoof-LSPs modes and Babinet’s principle^20^ is applicable to both the fundamental and high-order spoof-LSPs modes. By using full-wave numerical simulations and near-field imaging technique, we directly observe mode profiles of these high-order spoof-LSPs modes and verify the complementarity of electric and magnetic fields between MSS and complementary MSS for all spoof-LSPs modes, as predicted by Babinet’s principle. We also show that, compare with the fundamental spoof-LSPs mode^19^, these high-order spoof-LSPs modes exhibit larger frequency shifts to the refractive index of surrounding materials at microwave frequencies. For this feature, we experimentally illustrate the potential application of the complementary MSS as a plasmonic sensor to detect the refractive index change of the surrounding material at microwave frequencies.

## Results

**Figure 1:**
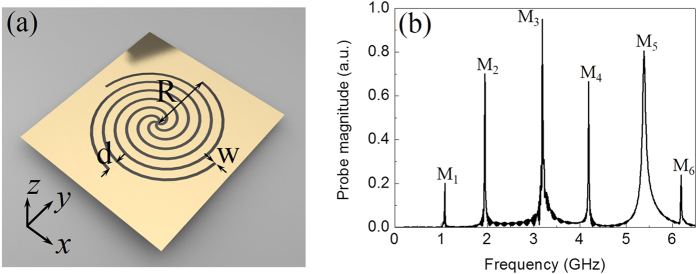
(**a**) The geometry of the complementary metallic spiral structure. The outer radius is *R* = 12.5 mm. The width of the spiral slots is *w* = 0.5 mm. The spacing between neighboring slots is *d* = 1.5 mm. (**b**) The simulated near-field response spectrum of the complementary metallic spiral structure. Six distinct resonance peaks are indicated as M_1_-M_6_, respectively.

Figure 1(a) shows the geometry of the proposed complementary MSS, which consists of four air slots wrapped 1.5 turns such that the whole structure has an outer radius *R* = 12.5 mm. Each spiral slot has a width *w* = 0.5 mm and the spacing of neighboring slots at the outer radius is *d* = 1.5 mm. The copper film has a thickness of 0.018 mm. The dielectric substrate has a thickness of 0.2 mm with permittivity *ε* = 3.5. Note that similar plasmonic spiral structures^21^,^22^ also have been used to focus light into a subwavelength spot. To evaluate the spoof-LSPs resonances on the complementary MSS, we calculate its near-field response spectrum using a full-wave commercial software CST Microwave Studio. Since metals in the microwave region are treated as perfect electric conductors (PECs), the copper film is modeled as perfect electric conductors (PECs) in this study. Under the excitation of a monopole source (dipole current), Fig. 1(b) illustrates the simulated near-field response spectrum when the probe is located at the opposite side to the source and 1 mm above the complementary MSS. From the simulation results, we observe that apart from the previously reported two fundamental spoof-LSPs modes (M_1_ and M_2_)^17^,^19^, another four high-order spoof-LSPs modes (M_3_-M_6_) are emerged in the near-field response spectrum, similar to the high-order spoof-LSPs mode supported on MSS^18^.

**Figure 2:**
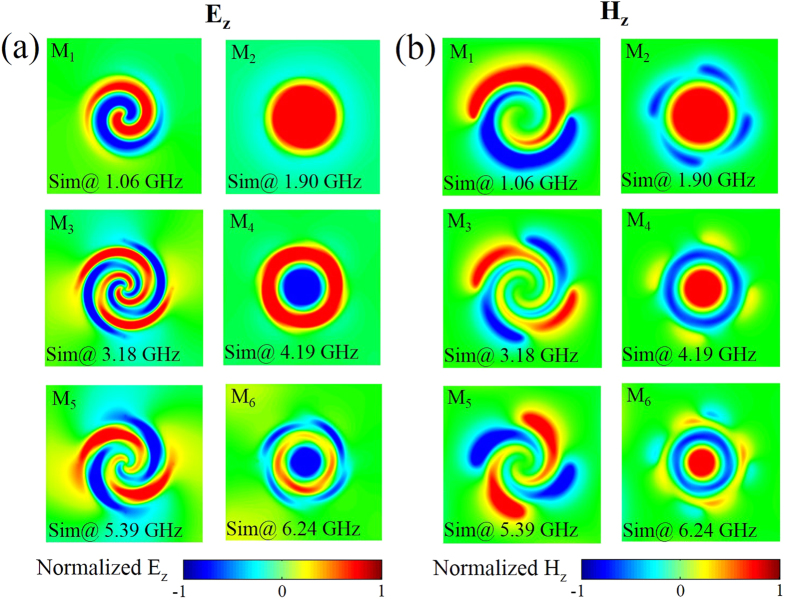
(**a**) The simulated near-field patterns of vertical electric field (E_z_) on a transvers *x-y* plane 1 mm above the upper complementary MSS surface for the fundamental mode (M_1_) at 1.06 GHz, the second-order mode (M_2_) at 1.90 GHz, the third-order mode (M_3_) at 3.18 GHz, the fourth-order mode (M_4_) at 4.19 GHz, the fifth-order mode (M_5_) at 5.39 GHz, and the sixth-order mode (M_6_) at 6.24 GHz, respectively. (**b**) The simulated near-field patterns of vertical magnetic field (H_z_) on a transvers *x-y* plane 1 mm above the upper metallic spiral surface for all modes as in (**a**).

To further explore the nature of these spoof-LSPs modes governing the electromagnetic response of the complementary MSS, Fig. 2(a,b) show the near electric field and magnetic field patterns for the six resonance peaks (M_1_-M_6_) identified in Fig. 1(b). Specially, the *z*-component of electric field [Fig. 2(a)] and magnetic field [Fig. 2(b)] is plotted within the *x-y* plane 1 mm above the complementary MSS upper surface. In all panels, the color scale has been saturated to show clearly the mode profile, ranging from red (positive) to blue (negative). Similar to the fundamental spoof-LSPs modes previously reported for complementary MSS^19^, these plots show that the first resonant mode (M_1_) is due to the magnetic LSPs, while the second peak (M_2_) emerges from the electric LSPs. Moreover, for all six spoof-LSPs modes (M_1_-M_6_), the electric/magnetic mode profiles on the complementary MSS [Fig. 2(a,b)] are exactly the corresponding magnetic/electric mode profiles on the MSS^18^, as predicted by Babinet’s principle^20^. The complementarity of electric and magnetic fields in the near-field region between a metallic structure and its complementary structure provides a convenient way to indirectly map the magnetic near-field of a metallic structure by measuring the electric near-field of its complementary structures^19^.

**Figure 3:**
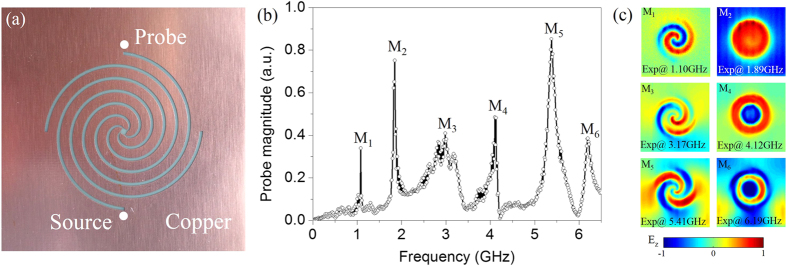
(**a**) The photograph of the fabricated complementary metallic spiral structure (thickness 0.018 mm) printed on a dielectric substrate (FR4) with thickness 0.2 mm. The monopole source and probe are indicated by a pair of white dots. (**b**) The measured near-field response spectrum of the complementary metallic spiral structure. The measured six distinct resonance peaks are indicated as M_1_-M_6_, respectively. (**c**) The measured near-field patterns of vertical electric field (E_z_) on a transvers *x-y* plane 1 mm above the upper complementary MSS surface for the first-order mode (M_1_) at 1.10 GHz, second-order mode (M_2_) at 1.89 GHz, third-order mode (M_3_) at 3.17 GHz, fourth-order mode (M_4_) at 4.12 GHz, fifth-order mode (M_5_) at 5.41 GHz, and sixth-order mode (M_6_) at 6.19 GHz, respectively.

We then proceed to demonstrate experimentally the predicted electric and magnetic resonance field patterns as well as the complementarity of electric and magnetic fields between MSS and complementary MSS. We fabricate an ultrathin (0.018 mm) complementary MSS via a standard printed circuit board fabrication process on a 0.2 mm-thick dielectric substrate (FR4) with relative permittivity *ε* = 3.5, as shown in Fig. 3(a). We place a transmitting monopole antenna 1 mm away from one side of the sample to excite the spoof-LSPs modes, and another receiving monopole antenna at the other side of the samples to detect the near-field response spectrum. The locations of monopole antennas are indicated by a pair of white dots in Fig. 3(a). Both antennas are connected to a vector network analyser (R&S ZVL-13). The measured near-field response spectrum of the complementary MSS is plotted in Fig. 3(b). In the measured spectrum, six distinct resonance peaks are clearly observed at frequencies *f*_1_ = 1.10 GHz, *f*_2_ = 1.89 GHz, *f*_3_ = 3.17 GHz, *f*_4_ = 4.12 GHz, *f*_5_ = 5.41 GHz, and *f*_6_ = 6.19 GHz, respectively.

Furthermore, we present in Fig. 3(c) the measured near-field distributions (E_z_) on the complementary MSS. By means of a near-field scanning techniques, we map the local *z* component of electric field of all six spoof-LSPs modes (M_1_-M_6_). Comparing Fig. 3(c) to the numerical results shown in Fig. 2(a), we conclude that the agreement between the simulation results and experimental images is satisfactory.

**Figure 4:**
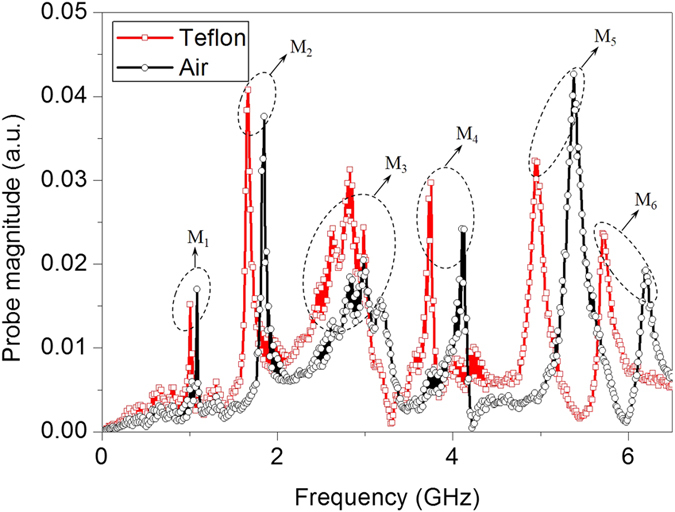
The measured near-field response spectra of the complementary metallic spiral structure covered by air (black line) and Teflon (red line), respectively. Much larger redshifts are observed for the high-order spoof-LSPs modes (M_3_-M_6_) than the fundamental spoof-LSPs modes (M_1_-M_2_).

The spoof-LSPs resonances of the complementary MSS are sensitive to the change of surrounding dielectric materials^19^. To illustrate this feature, in experiment we attach a dielectric sample (Teflon pad with refractive index n = 2.2) with thickness of 3 mm on the complementary MSS and measure the near-field response spectrum using the same experimental setup shown in Fig. 3(a). The measured near-field response spectra are shown in Fig. 4 when the complementary MSS is covered by air (black line) and Teflon pad (red line), respectively. From the measured results we observe that the higher order the resonant modes, the larger redshift the resonance frequencies. Specially, the redshift researches 0.078 GHz for the first-order mode (M_1_) and 0.182 GHz for the second-order mode (M_2_), while for the last four high-order modes, this value reaches 0.204 GHz for the third-order mode (M_3_) and 0.378 GHz for the fourth-order mode (M_4_), 0.432 GHz for the fifth-order mode (M_5_) and 0.477 GHz for the sixth-order mode (M_6_), respectively.

## Discussion

In conclusion, we have numerically and experimentally demonstrated that multiple high-order spoof-LSPs modes can be supported on complementary MSS at microwave frequencies. The complementarity of the near-field profiles between MSS and complementary MSS for all six resonant spoof-LSPs modes, as predicted by Babinet’s principle, has also been experimentally verified, which allows mapping the magnetic field distributions of magnetic spoof-LSPs mode on MSS by indirectly measuring the electric field distributions of the corresponding spoof-LSPs mode on complementary MSS. Moreover, we also show that high-order modes of complementary MSS exhibit larger frequency shift caused by the environmental refractive index change than the lower-order modes.

## Additional Information

**How to cite this article**: Gao, Z. *et al.* High-order spoof localized surface plasmons supported on a complementary metallic spiral structure. *Sci. Rep.* **6**, 24447; doi: 10.1038/srep24447 (2016).
